# Emerging trends and focus of giant cell tumor of bone research from 2001–2021: A visualization research

**DOI:** 10.3389/fonc.2022.1025876

**Published:** 2022-10-28

**Authors:** Ying-Song Han, Yi-Fan Yang, Gang Chen, Hai-Liang Yu, Zhi-Guo Zhang, Bin Zhou

**Affiliations:** ^1^ Department of Spine Surgery, Xiangtan Central Hospital, Xiangtan, China; ^2^ Department of Spine Surgery, The Second Xiangya Hospital, Central South University, Changsha, China

**Keywords:** bibliometrics, global trend, visualized study, giant cell tumor of bone, research focus

## Abstract

Giant cell tumor of bone is a highly invasive benign tumor with a high postoperative recurrence rate. Objective: This study aims to analyze the research hotspots and trends of global research on giant cell tumor of bone in the past 20 years, to provide a reference for relevant personnel in this field to carry out academic research. Methods: The literature related to giant cell tumor of bone from 2001 to 2021 was retrieved from the Web of Science. The bibliometrics research method and VOS Viewer were used to extract and analyze the keywords of the journal authors’ research institutions, and the research status and development direction in the recent 20 years were visualized. Results: A total of 2063 articles were included. The number of global publications is increasing every year. The United States contributes the most to global research, with the most citations and the highest H-index. The journal *Clinical Orthopaedics and Related Research* published the most articles on this issue. “Denosumab” and “h3f3a” will get more attention and be the next popular hotspot in the future. Conclusion: The study of giant cell tumor of bone is a hot spot of continuous development and has an important contribution to human health.

## 1 Introduction

Giant cell tumor of bone (GCTB) was described by Cooper and Travers in 1818 (1). The tumor is a rare mesenchymal tumor, which is classified as the intermediate tumor in World Health Organization (WHO) ‘s 2020 classification (2), that is, it is neither completely benign nor malignant due to recurrence (frequent) and lung metastasis (rare). They are made of mononuclear stromal tumor cells of (pre-) osteoblastic phenotype, mononuclear cells of the monocyte-macrophage lineage, and osteoclast-like multinuclear giant cells responsible for tumor osteolysis. Giant cell tumor accounts for 5% of all primary bone tumors and 20% of benign bone tumors (3, 4). According to the degree of local disease, various surgical methods were used for deterministic treatment. Surgical options may vary, from intra-focal curettage to extensive local resection, which can lead to significant morbidity in early adulthood. Surgery such as radiotherapy or embolization was the main treatment for unresectable diseases before the emergence of denosumab. However, these treatments are not sufficient to completely cure GCTB, and they have some shortcomings, such as radiation therapy achieving good local control but carrying the risk of malignant transformation. For embolization, repeat embolization is required. Denosumab therapy has a problem with regrowth after discontinuation (5–7). Recently, many studies on the mechanism, pathology, diagnosis, and treatment of giant cell tumor of bone have been published. However, the research on the qualitative and quantitative characteristics of giant cell tumor of bone as a whole is limited. It is necessary to evaluate the current status and trend of giant cell tumor of bone research and predict the promising popular topics and directions in this field.

Characterizing knowledge structure, studying the evolution of topics, and the emergence of topics have always been an important parts of information science. Bibliometric analysis is an important tool to evaluate the research activities and research trends of a specific topic. It is also the most prominent research trend in future research (8). Meanwhile, bibliometrics assists researchers in obtaining a large amount of information, which is applied for the evaluation of scientific research performance (9). In addition, collaborative networks between key researchers, countries, and leading research groups can be identified (10). Bibliometric analysis is also applied to make policy and clinical guidelines (11). Moreover, bibliometric analyses have been frequently applied in recent years (12) and the efficient analysis has been applied successfully to make studies more intuitional, including lung cancer (13), coronavirus (14), osteoarthritis (12), and subchondral bone (15).

This study aimed to assess emerging research trends through a bibliometric analysis of existing GCTB literature. The search results are based on the analysis of the SCI-EXPANDED to determine important research hotspots, and other basic indicators, such as the type of publications and the most outstanding writers, scientific journals, institutions, and countries that made a significant contribution to the topic This bibliometric study will help researchers to determine the potential research in the field of a hot spot and the latest trends.

## 2 Materials and methods

### 2.1 Data source

Publication information from the Web of Science (SCI-Expanded), which are considered as the optimal databases for bibliometrics (13).

### 2.2 Search strategy

All the published papers were collected from WoS and the database expiration date was set to 31 December 2021. In this study, the search terms were shown as follows: theme = giant cell tumor of bone AND publishing year = 2001–2021) AND Language = (English) AND Document types = (ARTICLE OR REVIEW). Additionally, the detailed information of certain countries or regions was refined by indexing country/region in the WoS.

### 2.3 Data collection

The publication criteria were shown as follows: (1) The manuscript focused on the theme of GCTB; (2) The document types are Article and Review. (3) The papers must be written in English. The exclusion criteria were also shown as follows: (1) The themes were not related to GCTB; (2) Articles were briefings, news, meeting abstracts, etc. All the records of publications, including the year of publication, title, authors’ names, affiliations, nationalities, abstract, keywords, and name of publishing journals were saved as.txt files from the WoS database and then imported into Excel 2019. In the last, GraphPad Prism 8.0 was used to analyze the data. Any problem had been solved by consulting experts to reach a consensus.

### 2.4 Bibliometric analysis

As was mentioned previously, the intrinsic function of WoS was to characterize the basic features of eligible publications. The H-index, indicating that a scholar has published H papers and they have been cited at least H times, was created to measure the impact of scientific research. Hence, it reflects both the number of publications and corresponding citations (16). In addition, relative research interest (RRI) was defined as the number of publications in one certain field by all field publications per year. The world map was performed by R software including python + numpy + scipy + matplotlib and the time curve of publications was depicted according to the previous article (9).

### 2.5 Visualized analysis

Visualization of Similarity viewer (VOS viewer) (Leiden University, Leiden, The Netherlands) is a software tool for constructing and visualizing bibliometric networks. These networks include journals, researchers, or individual publications, and they can be constructed based on citations, bibliographic couplings, co-citations, or co-authorship relationships. And CiteSpace was used to detect burst keywords for forecasting the possible hotspots and research frontiers in the future.

## 3 Results

### 3.1 Trend of global publications

#### 3.1.1 Amount of global publications

**Figure 1:**
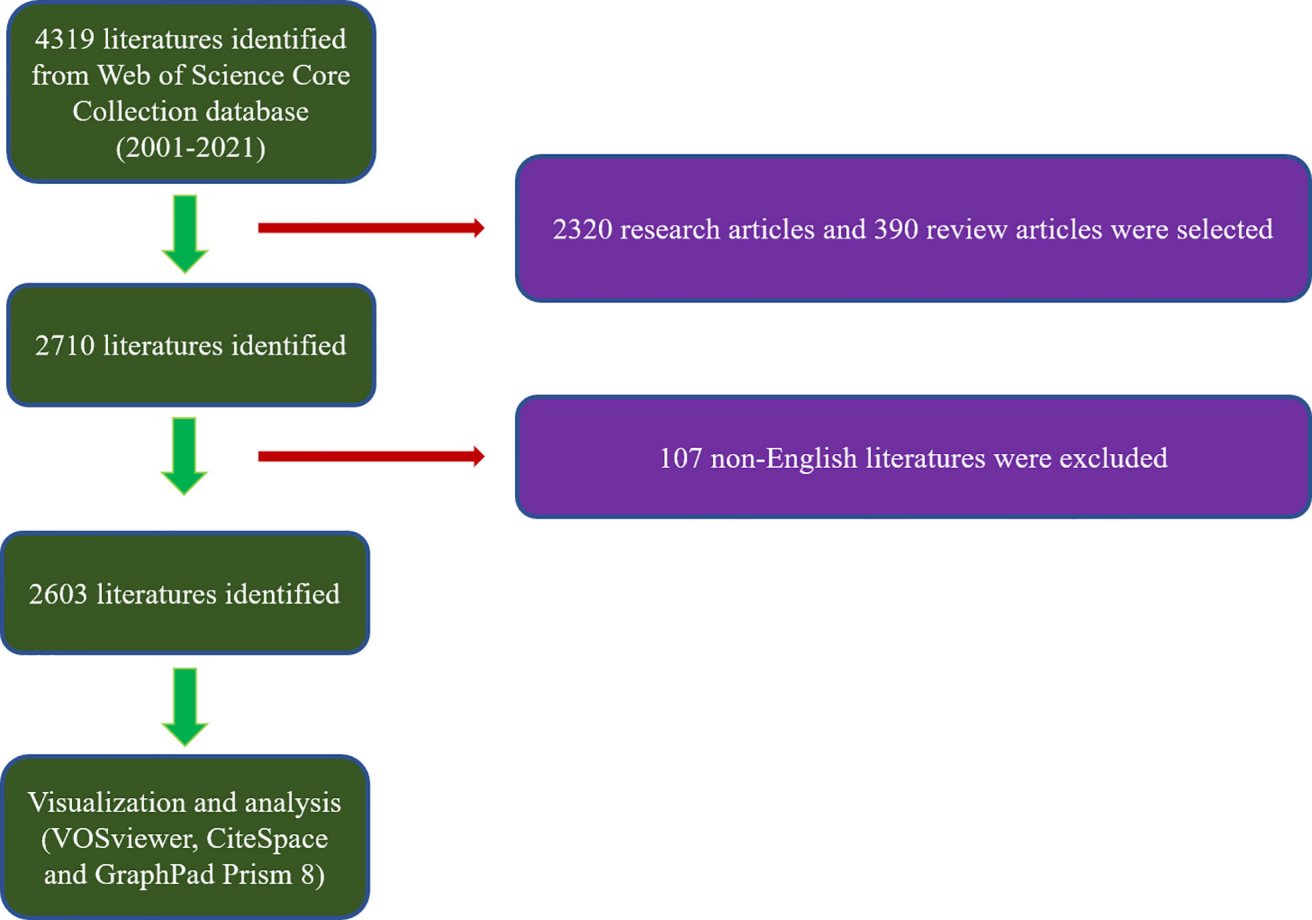
Flow diagram of the study identification and inclusion process.

**Figure 2:**
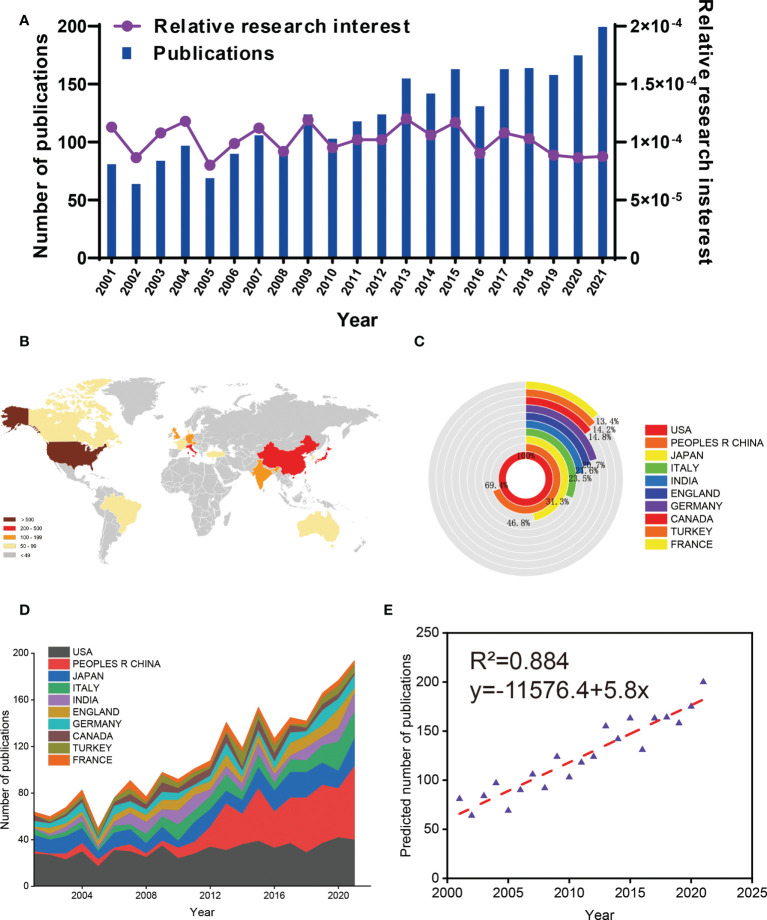
Global trends and countries/regions contributing to the research field of GCTB. **(A)** The annual number of publications and related research interests related to GCTB. **(B)** A world map depicting the distribution of GCTB. **(C)** The annual number of publications in the top 10 most productive countries/regions from 2001 to 2021. **(D)** The sum of GCTB-related publications in the top 10 countries/regions. **(E)** Model fitting curves of global trends in publications of/regions (R^2 =^ 0.884).

According to the search criteria, a total of 4319 articles on GCTB from 2001 to 2021 were collected. Finally, 2603 literatures identified were obtained for in-depth analysis (**Figure 1**). From 2001 to 2021, the trend of global publications generally increased year by year. In addition, relative research interest in the field has slightly declined over the past few years (**Figure 2A**).

#### 3.1.2 Contributions of countries and regions

In total, 82 countries and regions made contributions to publications in this field. Global article productivity is shown in **Figure 2B**. The United States reported the most papers (656, 25.20%), followed by China (455, 17.48%), Japan (307, 11.79%), Italy (205, 7.88%) and India (154, 5.92%) (**Figures 2C, D**).

#### 3.1.3 Global publications trend

In order to predict the future global publications trend, a logistic regression model was used to create a time curve of the number of publications. **Figure 2E** shows the model fitting curves of the growth trends to predict the number of global publications in the next few years.

### 3.2 Quality of publications of different countries and regions

#### 3.2.1 Total citation frequency

**Figure 3:**
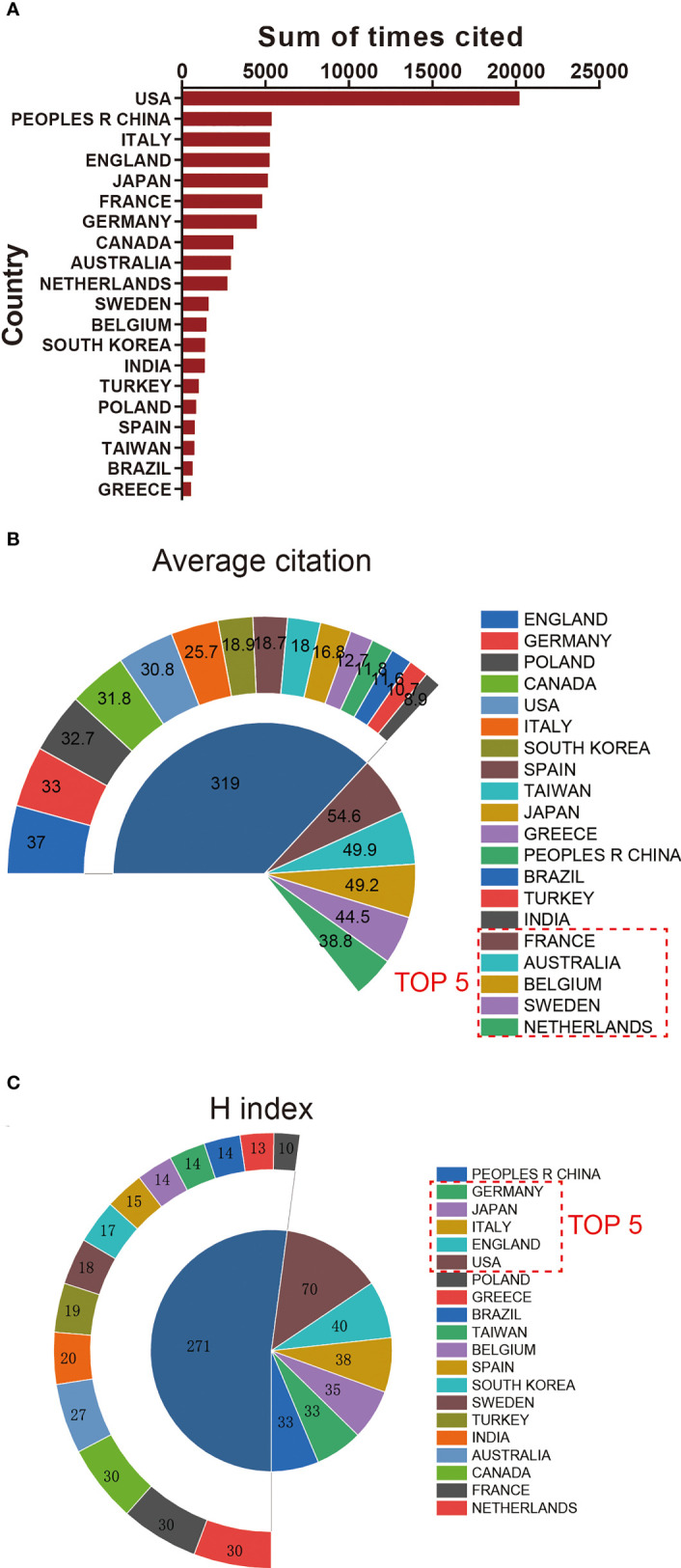
Total citation, H index, and citation frequency levels of different countries/regions. **(A)** The top 20 countries/regions of total citations of giant cell tumor of bone. **(B)** The top 20 countries/regions of the average citations per paper of giant cell tumor of bone. **(C)** The top 20 countries/regions of the H-index of organoids in giant cell tumor of bone.

In regards to WOS database analysis, we tallied the total citation frequency, average citation, and H-index of each country. Publications from the United States had the highest total citation frequency (20092). China ranked second in total citation frequency (5340), followed by Italy (5246), England (5197), and Japan (5094) (**Figure 3A**).

#### 3.2.2 Average citation frequency

Publications from France had the highest average citation frequency (54.6). Australia ranked second in average citation frequency (49.9), followed by Belgium (49.2), Sweden (44.5), and Netherlands (38.8) (**Figure 3B**).

#### 3.2.3 H-index

The relative publications from the USA had the highest H-index (70), followed by England (40), Italy (38), Japan (38), and Germany (35) (**Figure 3C**).

### 3.3 Analysis of global publications

#### 3.3.1 Authors

**Figure 4:**
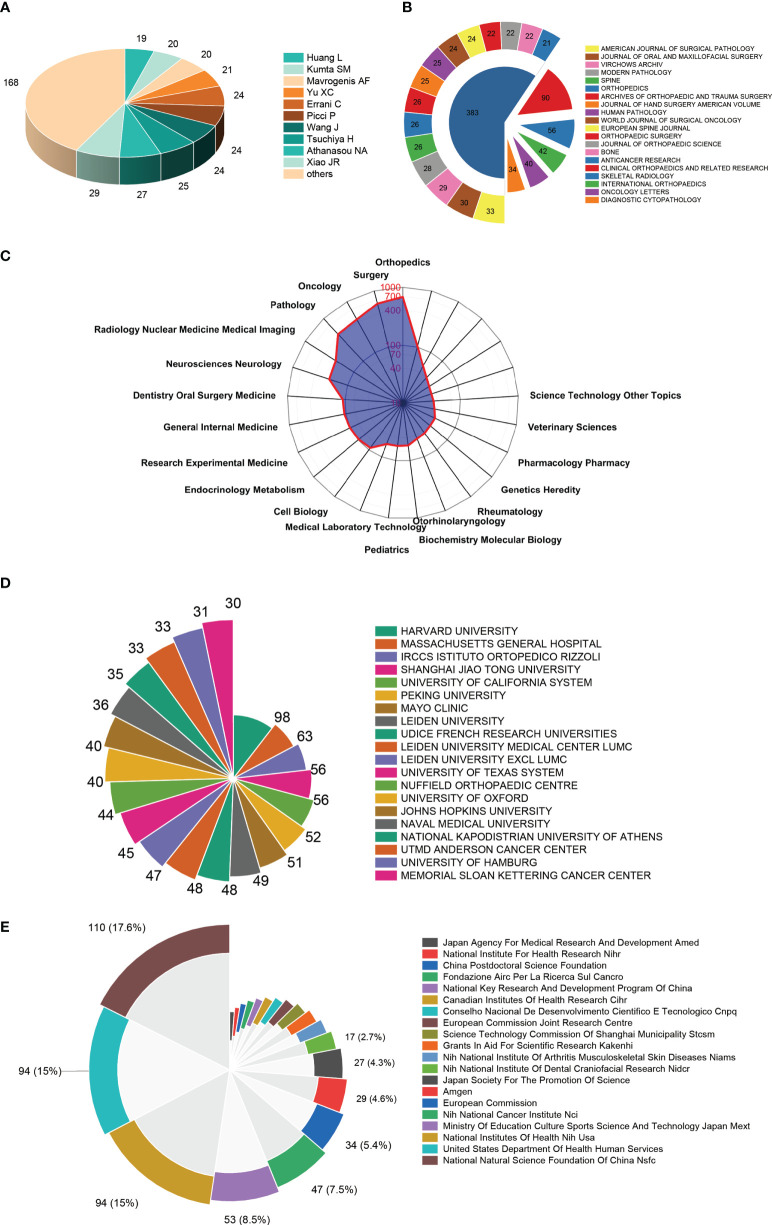
High-contribution authors, journals, research orientations, institutions, and funds of global publications about GCTB. **(A)** The top 10 authors with the most publications on GCTB. **(B)** The top 20 journals with the most publications on GCTB. **(C)** The sum of research orientations in the world. **(D)** The top 20 institutions with the most publications on GCTB. **(E)** The top 20 funding sources with most publications on GCTB.

There were 10324 authors in 2063 articles. The top 25 authors contributed a total of 475 publications, which accounted for 18.25% of all publications in this field. Xiao JR published the most research with 29 publications, followed by Athanasou NA with 27 publications and Tsuchiya H with 25 publications (**Figure 4A**).

#### 3.3.2 Journal analysis

About Individual Journals, *CLINICAL ORTHOPAEDICS AND RELATED RESEARCH* published the largest number of GCTB (90 publications). There were 56 publications in *SKELETAL RADIOLOGY*, 42 publications in *INTERNATIONAL ORTHOPAEDICS*, 40 articles in *ONCOLOGY LETTERS*, and 34 publications in *DIAGNOSTIC CYTOPATHOLOGY*. The top 25 journals with the most publications were listed in **Figure 4B**.

#### 3.3.3 Research orientations

**Figure 4C** shows the research direction distribution of GCTB-related literature. The most popular areas of research are orthopedics, surgery, oncology, pathology, radiology nuclear medicine medical imaging.

#### 3.3.4 Top productive institutions

The top 25 contributive institutions were listed in **Figure 4D**. HARVARD UNIVERSITY published the most (98 publications), MASSACHUSETTS GENERAL HOSPITAL ranked second (63 publications), while IRCCS ISTITUTO ORTOPEDICO RIZZOLI ranked third (56 publications).

#### 3.3.5 Funding sources

The top 25 funding sources were shown in **Figure 4E**. In totally, 110 publications were funded by the National Natural Science Foundation of China (NSFC) (ranked first), 94 publications were funded by the National Institutes of Health (NIH) (ranked second) and 94 publications were funded by the United States Department of Health Human Services (tied for second).

### 3.4 Bibliographic coupling analysis

#### 3.4.1 Journal:

**Figure 5:**
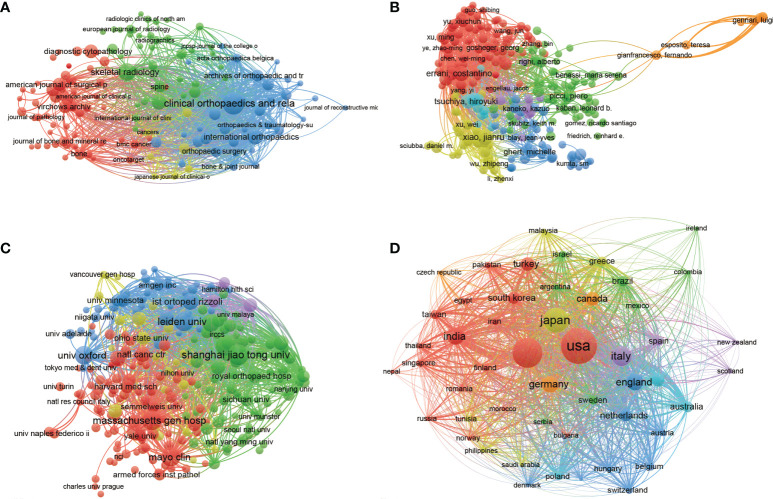
Mapping of bibliographic coupling analysis related to GCTB. **(A)** Mapping of the 134 identified journals on GCTB. **(B)** Mapping of the 229 authors on GCTB. **(C)** Mapping of the 231 institutions on GCTB. **(D)** Mapping of the 50 countries on GCTB. The line between different points represents that the journals/authors/institutions/countries had established a similarity relationship. The thicker the line, the closer the link between the journals/authors/institutions/countries.

The bibliographic coupling was used to analyze the similar relationship between documents. Firstly, we used VOS viewer to analyze the name of journals in total publications. There are 134 identified journals that appeared in total link strength which were shown in **Figure 5A**. The top 5 journals with larger total link strength were shown as follows: *Clinical Orthopedics and Related Research* (total link strength = 72865 times), *International Orthopaedics* (total link strength = 30093 times), *Orthopedics* (2021, total link strength = 22022 times), *Journal of Bone and Joint Surgery American Volume* (total link strength = 19739 times) and *Skeletal Radiology* (total link strength = 19111 times).

#### 3.4.2 Author:

Publications (defined as the minimum number of documents of an author more than 5) were produced by 229 authors and were further analyzed by the VOS viewer. As shown in **Figure 5B**, the top five authors with large total link strength were the following: Errani C (total link strength = 61641 times), Tsukamoto S (total link strength = 49242 times), Mavrogenis AF (total link strength = 48113 times), Xiao JR (total link strength = 44801 times), Gelderblom H (total link strength = 34315 times).

#### 3.4.3 Institution:

Papers (defined as the minimum number of documents of an organization that were used more than 5 and the maximum number of organizations per document no more than 25) were identified in the 231 institutions and analyzed using VOS viewer (**Figure 5C**). The top 5 institutions with largest total link strength were shown as follows: Leiden University (total link strength = 72502 times), Massachusetts General Hospital (total link strength = 57601 times), Shanghai Jiao Tong University (total link strength = 54360 times), Nara Medical University (total link strength = 47610 times), and Second Military Medicine University (total link strength = 47278 times).

#### 3.4.4 Country:

Publications (defined as the minimum number of documents of a country more than 5) originating from 50 countries were analyzed *via* VOS viewer (**Figure 5D**). The top 5 countries with large total link strength were as follows: USA (total link strength = 408200 times), China (total link strength = 337798 times), Japan (total link strength = 215168 times), Italy (total link strength = 203441 times) and England (total link strength = 133556 times).

### 3.5 Co-citation analysis

#### 3.5.1 Authors

**Figure 6:**
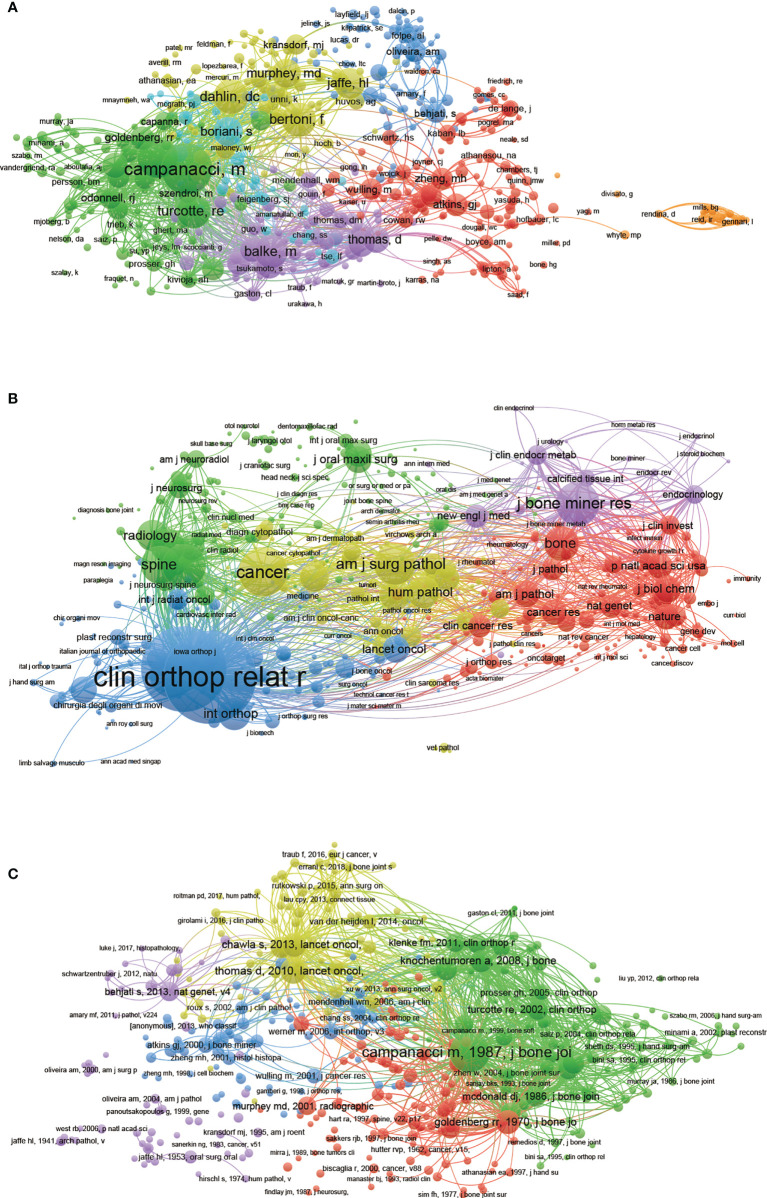
Mapping of co-citation related to GCTB. **(A)** Mapping of the co-cited authors related to this field. (The 555 points with different colors represent the 555 identified authors.) **(B)** Mapping of the co-cited journals related to this field. (The 573 points with different colors represent the 573 identified journals.) **(C)** Mapping of the co-cited references related to this field. (The 382 points with different colors represent the 382 cited references.). The point sizes represent the citation frequency. The line between different points indicates that they were cited in one paper. The shorter the line, the closer the link between two papers. The same color of the points represents the same research area they belong to.

The co-citation analysis was to consider the relatedness of the items based on the numbers they were co-cited. A total of 555 authors with a minimum 20 documents were analyzed using VOS viewer (**Figure 6A**). The top 5 publications with the largest total link strength were as follows: Campanacci, M (total link strength = 14589 times), Enneking, WF (total link strength = 9062 times), Balke, M (total link strength = 8988 times), Turcotte, RE (total link strength = 8211 times), and Bertoni, F (total link strength = 7584 times).

#### 3.5.2 Journals

The names of journals of co-citation analysis were performed using VOS viewer and the journal with a minimum number of citations over 20 were defined. As illustrated in **Figure 6B**, 573 journals were shown in the total link strength. The top 5 journals with the greatest total link strength were as follows: *Clinical Orthopaedics and Related Research* (total link strength = 173297 times), *Journal of Bone and Joint Surgery American Volume* (total link strength = 147999 times), *Journal of Bone and Mineral Research* (total link strength = 94818 times), *Cancer* (total link strength = 80186 times), and *Skeletal Radiology* (total link strength = 64934 times).

#### 3.5.3 References:

382 references (defined as minimum number of citations of a cited reference that were used more than 20) were analyzed *via* VOS viewer (**Figure 6C**). The top 5 articles with greatest total link strength were as follows: Campanacci, 1987, j bone joint surg am (17),(total link strength = 7000 times); Thomas d, 2010, lancet oncol (18), (total link strength = 3712 times); Odonnell rj, 1994, j bone joint surg am (19), (total link strength = 3417 times); Knochentumoren a, 2008, j bone joint surg am (20), (total link strength = 3413 times); Chawla s, 2013, lancet oncol (21), (total link strength = 3401 times).

### 3.6 Co-authorship analysis

#### 3.6.1 Authors:

**Figure 7:**
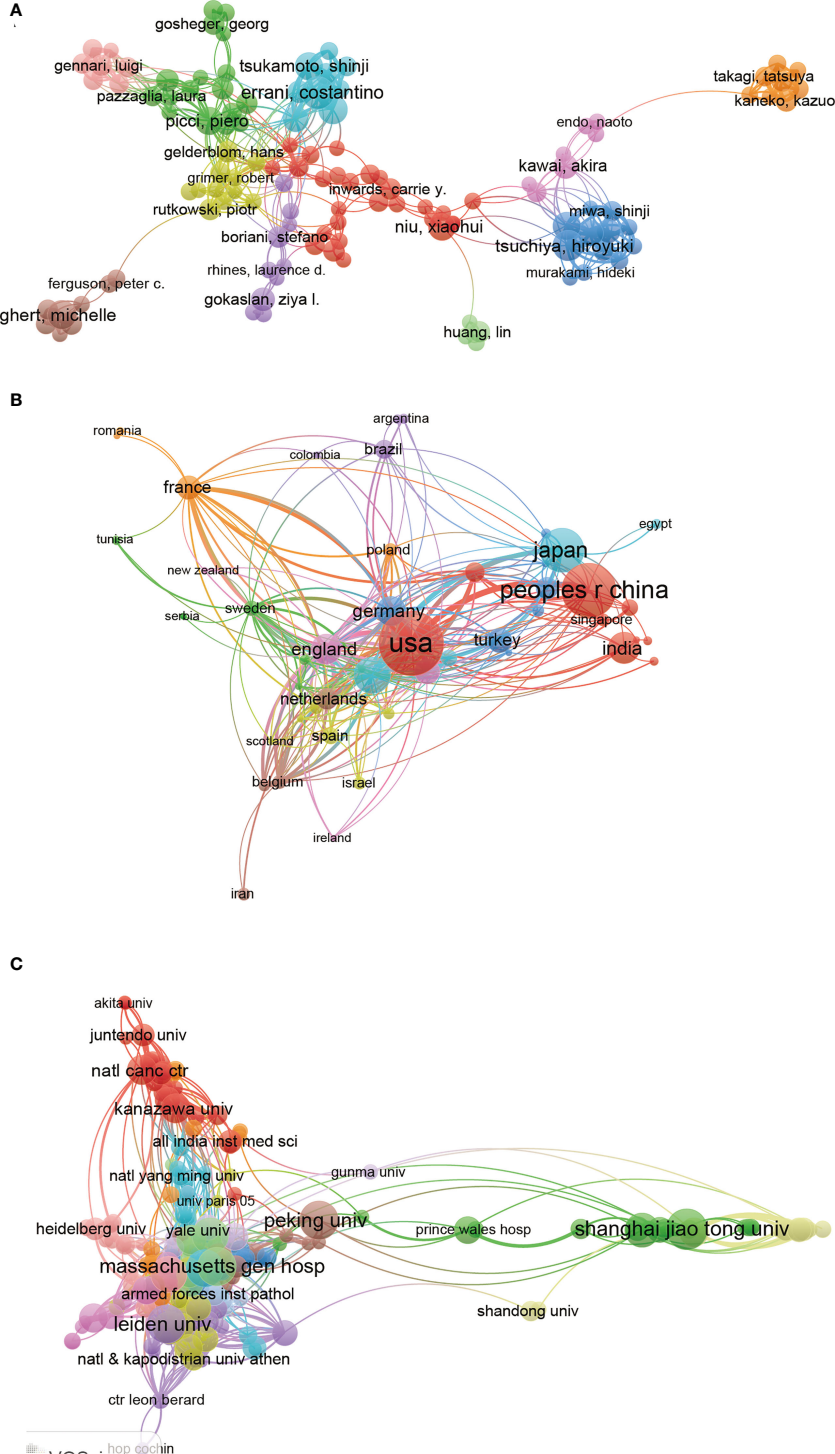
Visualized images of co-authorship analysis of global research about GCTB. **(A)** Mapping of the 119 author co-authorship analysis on GCTB. **(B)** Mapping of the 50 country co-authorship analysis on GCTB research. **(C)** Mapping of the 231 institution co-authorship analysis on GCTB research.

Co-authorship analysis was performed to evaluate the item’s relatedness based on the total number of co-authored papers. There are 229 authors with over 5 documents that analyzed using VOS viewer and the results were shown in **Figure 7A**. The top 5 authors with larger total link strength were as follows: Xiao, JR (total link strength = 116 times), Tsuchiya, H (total link strength = 101 times), Takeuchi,A (total link strength = 94 times), Errani, C (total link strength = 86 times), Yamamoto, N (total link strength = 82 times).

#### 3.6.2 Countries:

There are 50 countries with over 5 papers that were chosen and analyzed using VOS viewer and the results were depicted in **Figure 7B**. The top 5 countries with greatest total link strength were the following: USA (total link strength = 312 times), Italy (total link strength = 210 times), England (total link strength = 185 times), Germany (total link strength = 92 times) and Netherlands (total link strength = 92 times).

#### 3.6.3 Institutions:

There are 231 institutions with more than 5 documents were analyzed through VOS viewer (**Figure 7C**). The top 5 institutions with the greatest total link strength were shown below: Massachusetts gen hosp (total link strength = 114 times), Leiden univ (total link strength = 75 times), Amgen inc (total link strength = 61 times), Ist ortoped rizzoli (total link strength = 58 times), Mayo clin (total link strength = 58 times).

### 3.7 Co-occurrence analysis

**Figure 8:**
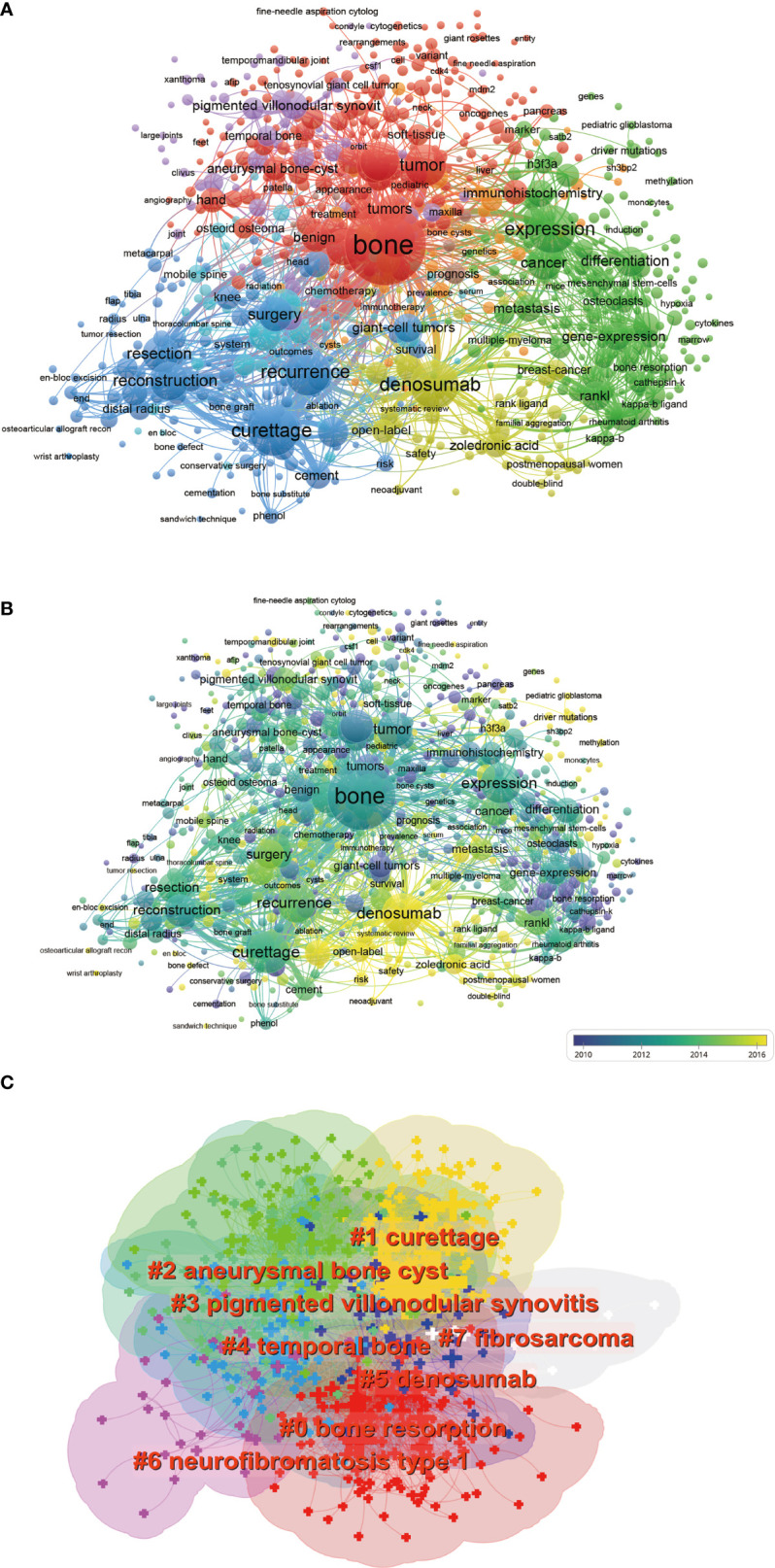
Visualization of co-occurrence analysis based on GCTB. **(A)** Mapping of keywords in the research on GCTB during 2001–2021; the frequency is represented by point size. **(B)** Distribution of keywords according to the mean frequency of appearance. Keywords in purple appeared earlier than those in green and yellow-colored keywords appeared later. **(C)** The keywords clustering knowledge map of GCTB during 2001–2021. Tag # was assigned to the cluster, and the smaller the number is, the more keywords are in the cluster.

The objective of co-occurrence analysis is to investigate popular directions and areas of researches, and it also plays a vital role in monitoring the developments in scientific research. Keywords, which were defined as the words used more than 5 times in titles/abstracts in all papers, were chosen and analyzed *via* VOS viewer. As shown in **Figure 8A**, the 789 identified keywords were roughly classified into 4 clusters. In the center of the co-occurrence map, the keywords, including bone, tumor, expression, denosumab, and curettage were shown more prominently with higher weight. Thus, further high-quality studies on GCTB in these directions are still required. Additionally, keywords were coded with different colors by the VOS viewer based on the average times they appeared in all the published papers (**Figure 8B**). The color purple meant that the keywords appeared earlier, whereas the color green and yellow indicated later appearance. According to the results, denosumab may be the next popular topic in this field. There were 8 clustering patterns in the research field of the GCTB, which are shown in the keyword clustering knowledge map (**Figure 8C**). “bone resorption” was the largest cluster (#0), followed by “curattage” (#1), and “aneurysmal bone cyst” (#2).

### 3.8 Focus shift and research frontiers

**Figure 9:**
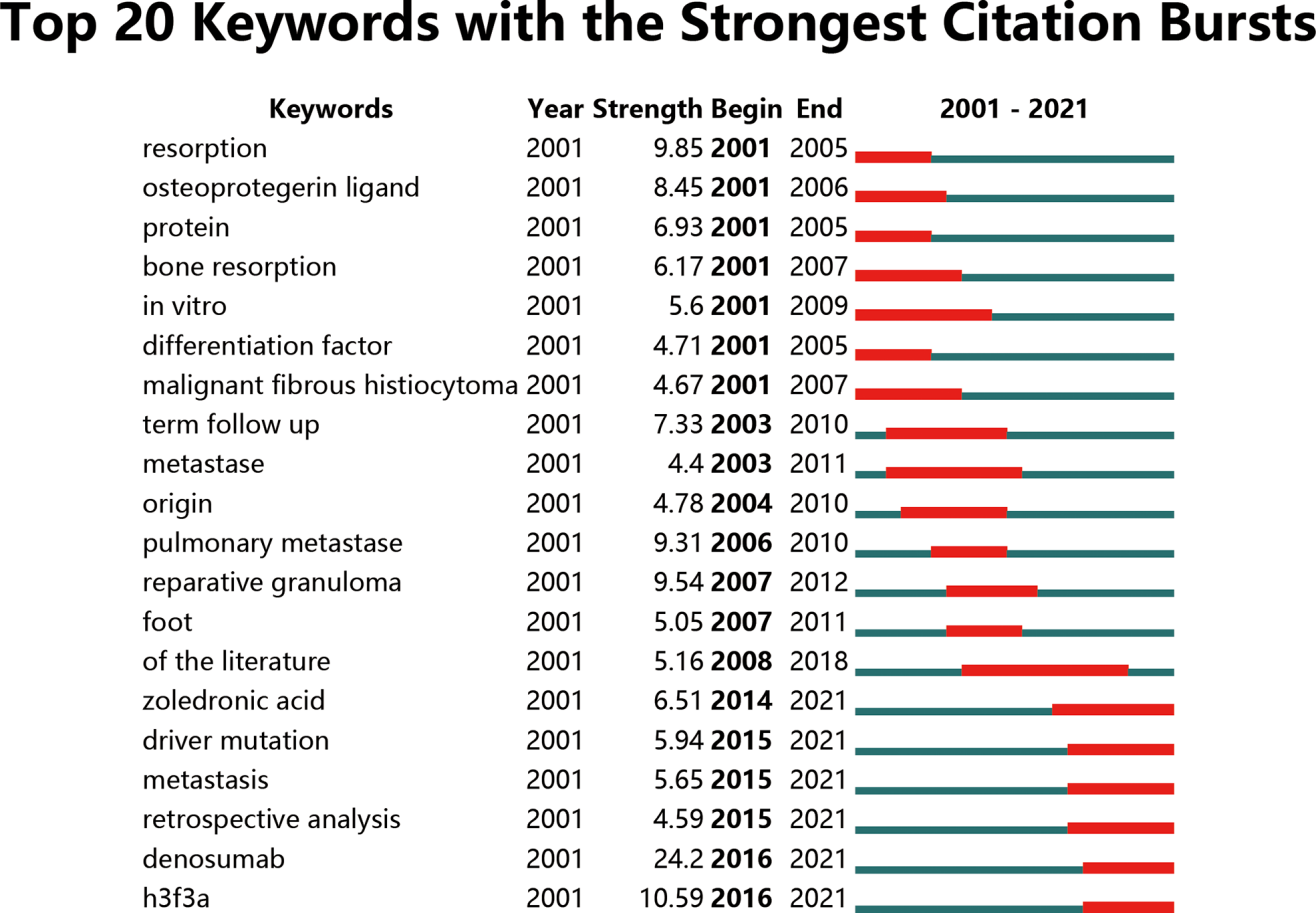
Top 20 keywords with the strongest citation bursts. Keywords with the strongest citation bursts in the scientific literature were analyzed and visualized in the keyword’s bursts map. The lines in red stood for the burst detection years. Keywords with red lines extending to the latest year can indicate the research frontiers in a short period of time in the future.

Keywords with intense bursts in a short period can act as a sensitive indicator to reflect the research focus. Recent burst keywords provide researchers the possible research frontiers in the short future. A keyword burst map was generated by CiteSpace, where the strength and the beginning or ending year of the burst was shown (**Figure 9**). The strength reveals the burst intensity, and the burst year indicates the transformation of the research focus and its duration. Early studies focused on the clinical manifestations (resorption begin in 2001) and biomarkers (osteoprotegerin ligand begin in 2001 and protein begin in 2001) of GCTB. Afterward, malignant fibrous histiocytoma (begin in 2001), metastasis (begin in 2003), reparative granuloma (begin in 2007), and zoledronic acid (begin in 2014) generalized the research focus transformation over the past 21 years. In recent years, driver mutation (begin in 2015), denosumab (begin in 2016), and h3f3a (begin in 2016) appeared and kept bursting till 2021.

## 4 Discussion

GCTB studies are very meaningful for patients to reduce the recurrence rate and financial burden. To our knowledge, this study is the first systematic bibliometric assessment of scientific publications on GCTB from 2001 to 2021. We aimed to obtain an overview from two perspectives to outline the GCTB research field. One is the general characteristics, including publication numbers, contributed countries, core journals, etc. Another is the substance contents, including developmental skeletons, research frontiers, etc.

### 4.1 Trends in GCTB research

Recently, the progress of giant cell tumor of bone has become an exciting and steady developing research field (22, 23). As shown in this study, there has been a steady increase in the number of publications produced each year. Although there has been a decline in research interest over the past few years, we predict a steady increase in the number of publications in the future based on current data (**Figure 2**). As a result, more in-depth studies on GCTB will be published in the coming years. The current optimistic results will also allow researchers to conduct further high-quality studies.

### 4.2 Quality and status of global publications

The total number of citations and H index of a country represents the academic influence and publication quality of that country (24). The United States makes the greatest contribution to global research in terms of the total number of published papers, total citation frequency, and H index (**Figure 3**). Therefore, the United States can be regarded as a leader in this field.

Authors who have published more research in this field are shown in **Figure 4A**. This shows that further research by these authors can be paid close attention to obtain the latest progress of GCTB. More research on GCTB has been published in *Clinical Orthopaedics and Related Research*, *Skeletal Radiology*, and *International Orthopaedics.* The journals in the list (**Figure 4B**) may be the main publishing channels for future discoveries in this field. Further research in this area may appear at the top of the list. The top 3 research institutes with the largest number of articles are the leading organizations in GCTB research, which is consistent with the leadership of the top three countries in global publications.

In this study, a similar relationship among journals, institutions, and countries is established through bibliographic coupling analysis. Bibliographic coupling occurs when two works cite a common third work in their bibliography. These data show that Leiden University is the most relevant institution. *Clinical Orthopedics and Related Research* is the most relevant journal, while the USA is the leading country in this field. As a quantitative research method in bibliometrics, journal co-citation analysis can reveal the correlation between journals, authors, and references. The current results show that the milestone research in GCTB has a large total co-citation frequency. *Clinical Orthopedics and Related Research* is the most frequently co-cited journals in this field. With the development of science, scientific research cooperation has become an important factor to increase scientific research results. Scientific cooperation is of great significance to information exchange, knowledge dissemination, and resource sharing. Co-authorship analysis is used to assess cooperation among authors, institutions, and countries. Results with higher total link strength indicate that the countries/institutions/authors are more willing to cooperate with others.

### 4.3 The research focus of GCTB

The analysis of keywords can indirectly reveal various key research topics and characteristics in the research field. Based on co-occurrence analysis, we found the development direction and hot topic in this field. All the keywords of the papers were analyzed to create a map of the co-occurrence network. Four research directions can be observed from the co-occurrence map (**Figure 8A**). Although this result is consistent with common sense in this field, this study can make the future research direction clearer. At the center of the co-occurrence map, as is shown obviously, keywords including “bone”, “denosumab”, “expression” and “curettage”, etc. have a greater weight. The overlay visualization map was assigned colors by VOSviewer based on the average times the keywords appeared in the papers (25). This method is of great significance to the research direction of monitoring. The color bar indicates how fractions are mapped to colors. In the overlay visualization shown in **Figure 8B**, the color represents the year of publication. According to the results, “denosumab” (yellow color) may be the next hot topic in this field. The management of locally advanced and metastatic GCTB underwent a paradigm shift with the recognition of the role of receptor activators of the nuclear factor Kappa B ligand (RANKL) in the origin of the disease, and the discovery and subsequent trials of denosumab (RANKL inhibitor) in GCTB (26). These cases, which had previously been treated only with local therapy and had a high failure rate, are now managed by a multidisciplinary team that combines systemic therapy with local measures to improve outcomes (7). But the increase in the recurrence rate of dinolizumab treatment is still worrying. More research and large-scale clinical studies are needed.

Burst keywords reveal the research hotspots and their transformation from surgery to drug and advanced therapies. Particularly, burst keywords that continue to the present indicate the potential trends and possible frontiers in the field of GCTB (**Figure 9**). The latest burst keywords include “zoledronic acid”, “driver mutation”, “denosumab”, and “h3f3a”. So, studies on these aspects might indicate the frontier of the GCTB field.

## 5 Strengths and limitations

Although the present study evaluated the status and trends of studies about GCTB *via* bibliometric and visualized analyses, the following items about limitations have to be mentioned. English language studies were included based on the SCIE database of the Web of Science database. Non-English language literature could have been omitted, leading to language bias. Additionally, we only selected literature from the Web of Science database as the data source, so the selected literature was not comprehensive enough.

## 6 Conclusion

The present study showed the global status and trends in GCTB research. The USA was the largest contributor to studies and had the leading position in global research in this field. The journal Clinical Orthopaedics and Related Research had the most publications related to this issue. We can predict that more research about GCTB will be published in the coming years. The scientific cooperation network showed that cooperation between different countries and institutions has been sufficient. Particularly, new therapeutic agents and diagnoses, involving denosumab and h3f3a, will get more attention and be the next popular hotspot in the future. Additionally, the latest burst keywords also include “zoledronic acid”, “driver mutation”, “denosumab”, and “h3f3a”. In short, the study of the GCTB is an ongoing research hotspot and contributes to human health.

## Data availability statement

The original contributions presented in the study are included in the article/supplementary material. Further inquiries can be directed to the corresponding author.

## Author contributions

This work was conceived by BZ. Data was collected and downloaded by YH and Y-FY. Y-FY and GC helped to check and verify data as two independent investigators. The visualization work was performed by YH. The manuscript was written by YH and H-LY. Z-GZ helped to revise the manuscript and proposed constructive opinions. All authors contributed to the article and approved the submitted version.

## Acknowledgments

The authors would like to thank Xiangtan Central Hospital, and Central South University Library for supporting the work.

## Conflict of interest

The authors declare that the research was conducted in the absence of any commercial or financial relationships that could be construed as a potential conflict of interest.

## Publisher’s note

All claims expressed in this article are solely those of the authors and do not necessarily represent those of their affiliated organizations, or those of the publisher, the editors and the reviewers. Any product that may be evaluated in this article, or claim that may be made by its manufacturer, is not guaranteed or endorsed by the publisher.
